# A quasi-experimental intervention protocol to characterize the factors that influence the acceptance of new foods by infants: mothers’ diet and weaning method. Dastatuz project

**DOI:** 10.1186/s12889-021-10967-7

**Published:** 2021-05-13

**Authors:** Iratxe Urkia-Susin, Diego Rada-Fernandez de Jauregui, Estibaliz Orruño, Edurne Maiz, Olaia Martinez

**Affiliations:** 1 Department of Pharmacy and Food Science, Faculty of Pharmacy, University of the Basque Country EHU/UPV, Vitoria-Gasteiz, Basque Country Spain; 2 Bioaraba Health Research Institute, Nutrition and Food Safety group, Vitoria-Gasteiz, Araba, Basque Country Spain; 3 Department of Clinical and Health Psychology and Research Methodology, Faculty of Psychology, University of the Basque Country EHU/UPV, Donostia-San Sebastián, Gipuzkoa, Basque Country Spain; 4 Biodonostia Health Research Institute, Mental Health group, Donostia-San Sebastián, Basque Country Spain; 5 Department of Preventive Medicine and Public Health, Faculty of Pharmacy, University of the Basque Country EHU/UPV, Vitoria-Gasteiz, Basque Country Spain; 6 Bioaraba Health Research Institute, Araba University Hospital, Vitoria-Gasteiz, Araba, Basque Country Spain

**Keywords:** Children food neophobia, Fruits and vegetables, Dietary intervention, Early factors, Maternal diet, Breastfeeding, Baby-led weaning

## Abstract

**Background:**

Children usually refuse to eat and taste fruits and vegetables; and turning unhealthy eating habits around is an important social challenge in industrialized countries. The *Dastatuz* project aims to study children food neophobia and to enhance fruit and vegetable acceptance.

**Methods:**

A quasi-experimental, multicentre, controlled and prospective intervention study is proposed, in which early factors influencing new food acceptance will be studied. Mothers in the third trimester of pregnancy (*n* = 144) and their infants will be the study population. Experimental groups will be established based on mothers´ fruit and vegetable intake (standard or high intake) and weaning method (baby lead weaning vs spoon feeding). The project will assess the possible impact of maternal diet and complementary feeding on infants eating behaviour until 18 months of age. Outcome measures will comprise maternal diet and psychological features during pregnancy and breast-feeding (validated questionnaires). Compositional and physicochemical analysis of milk during breastfeeding will also be carried out. During weaning, until 18 months of age, children’s diet will be assessed with 24 h recalls and acceptance of new fruits and vegetables will be studied using video recording.

**Discussion:**

If the intervention is effective, this research work would have a high potential to be transferred to future public health programs or nutrition guidelines, as a feasible solution to achieve a higher intake of fruits and vegetables among children.

**Trial registration:**

This study is registered at ClinicalTrials.gov. Identifier: NCT04262102. Registration date: February 10, 2020 - Retrospectively registered.

## Background

Neophobia or new food refusal (ranging from severe rejection to a moderate manifestation) is a common behaviour in children and should be carefully considered due to the important implications on the current and future health status of the infants [1–3]. Parents, caregivers and health professionals are concerned about infant food neophobia and picky eating, since it can derive in an inadequate diet [4, 5]. The American Psychiatric Association described food neophobia as ‘avoidance and/or restriction to food based on the extreme sensitiveness to the appearance, colour, smell, texture, temperature or taste’ [6]. Hay et al. [7] described a 0,3% prevalence in the Australian population over 15 years of age and showed an association to socio-economic disadvantaged groups and obesity. Although there is limited prevalence data, it is known that clinically significant feeding and eating difficulties are common in early childhood, with estimates of 5–25% in young children [1, 8]. Neophobia has been linked to poor growth and deficient development [9, 10]. Moreover, childhood constitutes the key period in which feeding behaviour is established and then maintained up to adulthood [11, 12]. This is the reason why many authors highlight the relevance of addressing neophobic behaviour since childhood [13].

Fruits and vegetables (FV) are the main food target in infant food neophobia, because despite their relevant health contribution [14], these are the most commonly rejected foodstuffs and, consequently, they have become a health objective. Nutritional interventions and/or educational programs carried out in the past years have not succeeded to increasing FV intake further than half a portion [15]. According to the literature, fruits are more easily accepted by children than vegetables [16, 17]. There is still a compelling need for a more accurate characterisation of the children’s food consumption profile. National and local dietary surveys, such as the ENALIA Study [18] and the COSI Study [19], and unpublished data from the Vitoria-Gasteiz City Council Nutritional Observatory (2007) showed that only around 20–30% of children follow fruit intake recommendations and around 10% fulfil vegetable intake recommendations. In the view of the above mentioned data, it is plausible to think that FV intake figures will be even lower for neophobic children.

Norris, Spettigue and Katzman [20] pointed at the need for a better definition of factors affecting neophobia prevalence at different stages of life. Indeed, it would be useful to identify and characterize those early variables that might act as determinants of the neophobic behaviour prevention. There are limited prospective interventions conducted on the influence of mothers’ diet and complementary feeding at weaning on food neophobia or picky eating. One of the scarce works, a recent retrospective study based on online questionnaires, did not find any significant difference between complementary feeding method and eating behaviour or neophobia [21]. This observation opposes to the results of the intervention study carried on by Taylor et al. [22], in which an adapted baby-led weaning resulted in less food fussiness than spoon feeding. These aspects must be addressed taking into account the individual differences on food acceptance, such as caregivers eating habits, which, undoubtedly, influence food preference development in infants [17, 23].

Some authors have described a positive reaction of newborn children towards foods or substances consumed by their mothers during pregnancy or breastfeeding period [24–26]. Moreover, several studies have described lower levels of picky eating and/or food neophobia in children who have been exclusively breastfed for 6 months [27]. Some authors defend that breastfeeding itself improves acceptance of new foods, while some others consider that the variety of flavours present on the mother’s milk may constitute a key factor influencing future food acceptance [17, 27–31]. Nonetheless, there is an absence of empiric evidence to explain the causal relationship between breastfeeding and picky eating behaviour [3]. The relation between the diet and the flavour profile has been observed in mammals, but the sensory profile in human milk has only been studied recently [31, 32]. To the best of our knowledge, there is no research on the sensory profile of human milk related to the overall diet profile of the mother [29].

Regarding the weaning period, a new approach called Baby-Led Weaning (BLW), as opposed to the traditional spoon-feeding (SF), has become popular mostly in the United Kingdom and New Zealand [33]. Actually, regarding the Spanish state, a recent study carried out in Galicia shows a general estimated prevalence of BLW of 14% [34]. Although there is not a clear or precise definition, BLW can be characterized as follows: children are the ones feeding themselves, deciding which will be the rhythm and quantity of food to swallow, using their hands, ingesting pieces of food (not purée, or less than 10% of the total ingested food in purée), sharing family meals, and eating the same family-food [33, 35].

BLW has arisen some concerns on parents, caregivers and health professionals, mainly due to the chocking risk [36]. Nevertheless, some authors have reported no difference on the chocking risk between traditional weaning and BLW [37, 38]. Additionally, there is also a concern about the unfulfilment of the energy requirements of the child [33], particularly regarding initial energy intake. However, the available scientific evidence is not strong enough to support this idea [39]. Among nutrients, iron deficiency is the most generally stated worry [40]. Nonetheless, it has been demonstrated that BLW does not increase iron deficiency nor growth faltering [38]. In any case, children’s diet should be as balanced as possible, in order to fulfil all the nutritional requirements and avoid deficiencies. Some paediatricians have manifested their concern about the possible inappropriateness of food offered to the child when following BLW, stating that meals could be high in fat and too salty [39]. Moreover, allergen intake is also a subject of debate, because food selection is baby-led and this could imply less control over allergen intake in a period when children are more susceptible to food allergens [4]. However, some positive aspects have also been pointed out: usually parents following BLW fulfil WHO (World Health Organization) recommendations regarding the start of complementary feeding at 6 month of age, while SF is associated to an earlier weaning [35]. It has also been observed that when following BLW children develop a better ability for satiety responses [41], which constitutes a protection against obesity. Furthermore, BLW has been related to a greater food variety intake or acceptance [39].

Additionally to the development of FV liking and acceptance, it has been proved to be a malleable aspect [17]. Repeated exposure to certain FV or exposure variety seems to have an impact on acceptance. Maier et al. [24] also concluded that the frequency has more importance than the amount of FV offered. Experts point out that flavour/food repetition is relevant, providing a saturation limit is not exceeded [24, 28]. Finally, some factors, such as the texture, which could be important in the weaning period, remain understudied even though some authors have proved its relevance in food acceptance during the introduction of the complementary diet [42, 43].

In view of the above, and given the increasing popularity of the baby-led approach, the influence of the weaning method (traditional SF versus BLW) needs to be urgently addressed by means of a systematic methodology. Several methodological aspects should be specially considered, particularly paying attention to the food intake reports. In this regard, some authors have shown mismatching of parents/caregivers reported questionnaires and objective measurements [3, 44]. Taylor et al. [22] performed the only randomised controlled trial designed as such until now. The authors pointed out the relevance of proper and accurate information to parents about the correct dietetic approach for BLW. Along with that, a review demonstrated that educational interventions improve complementary feeding practices, including: exclusive breastfeeding duration enlargement, beginning of complementary feeding delay, offered foods adequacy and hygiene practices [45].

All the stages mentioned above (pregnancy, lactation and later caring stages of the baby) involve a huge change in women’s lives. That is why studies involving these stages should pay special attention to information and support given to families. In fact, pregnancy might be a source of stress [46], fatigue and has a negative impact on the quality of life, which might be associated to depression and anxiety [47, 48]. This period is full of expectations around birth and support given by their partners, relationship with healthcare professionals, active participation on decision making, and pain management [49]. On the other hand, the duration and exclusivity of breastfeeding has been predicted by the variable breastfeeding self-efficacy [50]. In order to persist and achieve concrete objectives it is fundamental that progenitors believe they are capable of becoming “good parents” [51]. Although the relationship between psychological health and attachment bond has vastly been studied [52, 53], investigations assessing the possible association between psychological wellbeing/perceived self-efficacy and parental stress of the mother and the decisions taken regarding the baby’s feeding remain unknown. Nonetheless, those feelings could influence the feeding modalities employed by parents, and it is becoming clear that control-based parental feeding styles affect negatively the children’s eating performance, while modelling positive eating behaviour happens to be successful [54–57].

With the aforementioned aspects in mind, we propose an intervention protocol to study the influence of the mother’s diet and the weaning method, as possible early factors, conditioning new fruit and vegetable acceptance during the period in which these foods are introduced in the child’s diet. The protocol has been designed taking into account the psychological aspects involved in the stages studied.

## Methods

### Hypothesis

A high intake and variety of FV during pregnancy and breastfeeding will lead to distinctive sensorial experiences for the baby, different from the ones whose mothers followed a “standard diet” (SD) (as described in the National Nutrition Surveys). This consumption profile will promote the acceptance of FV along complementary feeding. Additionally, a correct BLW may also contribute to establish healthy eating habits in children.

### Objectives

The main objectives of the present study are: on the one hand, to describe the possible impact of the mother’s diet during pregnancy and breastfeeding on children’s eating behaviour from weaning until 18 months of age; and, on the other hand, to study the possible effect of the weaning method (spoon-fed or baby-led) on the eating behaviour of children from weaning until 18 months of age.

This implies the following secondary objectives which are: (i) to analyse whether there is a relation between the mothers’ diet and the physicochemical characteristics of human milk, (ii) to study the possible relation between the mothers’ diet patterns and the chosen weaning style, (iii) to examine the relationship between the mothers’ psychological well-being, self-efficacy, parental stress and decision making regarding the child’s feeding, (iv) to characterise the BLW diet versus the SF diet regarding nutrient intake and main health indicators of children during the first 18 months of age, and (v) to carry out a risk assessment of potential food allergen intake associated to the BLW diet versus the SF diet.

### Design

The *Dastatuz* project (named after the Basque word for “tasting”) is a quasi-experimental multicentre, controlled prospective intervention study. Mothers in the third trimester of pregnancy (*n* = 144) and their infants constitute the study population. Mothers in the second trimester of pregnancy will be recruited for the study. Participants will be assigned to different diet patterns or to different weaning method groups according to their own food likings and caregivers´ willingness to perform weaning following one or other approach. A non-randomised design was chosen in order to replicate a realistic scenario which could be easily reproduced in future. Guidelines from the TREND statement for non-randomised control trials on behavioural and public health interventions will be followed meticulously.

### Participants

Women will be selected through the midwife consultation from the Basque Primary Healthcare Units (Northern Spain) on the second trimester of pregnancy attending routine control consultations. Midwifes will offer information regarding the project to the future mothers who then can directly contact the researchers in case they are willing to participate in the study.

The inclusion criteria will be: healthy mothers (aged between 18 and 45 years) who are notified about the study (and express interest to participate) before week 24 of gestation; who could be Basque, Spanish or English speakers and will live in the Basque Country until the child will be 18 months old. The exclusion criteria include unhealthy mothers or complicated pregnancies (all types of diabetes mellitus, high blood pressure, etc.), those not willing to breastfeed, mothers with extreme diet restrictions and those with severe non-adherence to prescribed diets. Additionally, any conditions that may be contraindicated for the development of the project will also be excluded (i.e. post partum complications, diseases). After birth, women will be excluded if their infant is born before 37 weeks of gestation; or if a congenital abnormality, physical condition, or intellectual disability, which might affect the infant’s feeding or growth is identified. Finally, milk will not be collected from those breastfeeding mothers who are taking any medication, which might alter significantly milk composition.

### Sample size

Sample size (*n* = 144) has been estimated taking into account main variable: FV intake achievement (proportion of children who reach recommendations). Accepting an alpha risk of 0.05 and a beta risk of 0.2 in a bilateral contrast, 72 subjects in the first group and 72 in the second are required to detect as statistically significant the difference between the proportion of children who reach recommendations, which for the control group is expected to be 25% and for the treatment group 50%. The final sample size has been increased by 20% in order to accommodate a drop out of 20%.

### Allocation to groups

Assignment to the control or treatment groups will be conducted according to mothers likings, type of diet and willingness to perform the weaning process traditionally (SF) or following a baby-led approach (see Table 1). Group assignment will be blind for participants.

**Table 1:** Distribution of the control (Ctrl) and intervention (I) groups

Groups	Mothers´ diet during last trimester of pregnancy and breastfeeding	Weaning approach	Group codes
1	“Standard” diet as described in National Diet Surveys (Ctrl)	Spoon feeding (Ctrl)	SD_SF
2		Baby-led (I)	SD_BLW
3	High Fruit and vegetable intake (I)	Spoon feeding (Ctrl)	HFV_SF
4		Baby-led (I)	HFV_BLW

*SD* standard diet; *SF* spoon-feeding; *BLW* Baby-Led Weaning; *HFV* high fruit and vegetable

#### Intervention group

Baseline questionnaires and initial dietary assessment will be the base for group assignment. Once participants are matched to a group, they will be given a code and will be anonymously treated thereafter. Those above the national (Spain) mean consumption of fruits and vegetables (about 414 g/person/day [58]) will be included in the intervention group. During the study, participants will be encouraged and assessed to achieve even a higher FV intake, following the recommendations given by Aune and colleagues of 800 g/person/day [14]. If a significant decrease in FV intake is detected in the participants' diet during the follow-up visits, dietetic assessment will be performed in order to restore the initial intake. With regard to the weaning groups, those better disposed to follow a baby-led approach will be included in the intervention group.

#### Control group

Baseline questionnaires and initial dietary assessment will be the base for group assignment and the study participants assigned to the control group will receive the same anonymous treatment as those in the intervention group. Those below the national (Spain) mean consumption of fruits and vegetables (about 414 g/person/day) or/and those who claim to have difficulties achieving the recommended portions will be included in the control group. When a fruit consumption of one standard deviation below the mean is detected, dietetic assessment will be performed in order to encourage, at least, the mean intake. As for the weaning groups, those better disposed to follow a spoon-feeding approach will be included in the control group.

### Study procedure

**Fig. 1:**
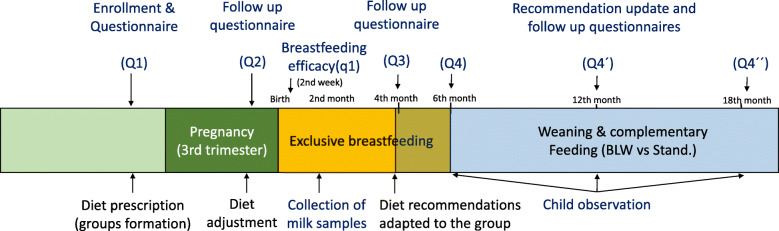
Timeline of the *Dastatuz* project. Legend: Main stages: 3rd trimester of pregnancy, breastfeeding and weaning and complementary feeding until the children are 18 months old. Q1, Q2, Q3, Q4, Q4’and Q4´´ refer to online questionnaires in each stage

Regardless of the group assignment, all participants will receive the current standard recommendations and care. Dietetic guidelines will be explained at the beginning of the study (last trimester of gestation) and after birth to ensure breastfeeding requirements. Intervention groups will receive additional diet recommendations so that they keep to the high fruit and vegetable consumption and to assure safety and a balanced diet along complementary feeding by BLW. The follow-up for the control group will be focused on ensuring a standard fruit and vegetable consumption and reinforcing general dietetic guidelines. Diets of mothers and children will be closely reviewed during the study to prevent deterioration. This implies a time lapse from the third trimester of the pregnancy until children are 18 months old. Follow-up, measurements will be performed on 6th–7th months of pregnancy, before delivery; on the 2nd, 4th, 6th, 12th and 18th months from birth. Figure 1 summarises the study outline.

Participants will receive information in 5–6 sessions throughout the study (with an approximate duration of 20–40 min) which will be delivered by a dietician-nutritionist. These meetings were planned to be held face to face, but they were adapted to online format due to the measures adopted within the COVID19 pandemic. Informative sessions will be either individual or group meetings (always considering participants allocation group) since the dates allocated for the sessions will depend on each participant’s delivery date: before the 3rd trimester of pregnancy, 1 month before the delivery date, and 4 months, 6 months, 12 months and 18 months after the delivery date. The topics that will be addressed in these meetings include: healthy eating, breastfeeding, complementary feeding, balanced diet, dietetic recommendations, technical aspects regarding completion of questionnaires, and signing of the informed consents. Additionally, instructions on different study procedures will also be given (e.g. milk extraction, child observation).

### Outcomes

#### Primary outcomes

**Fig. 2:**
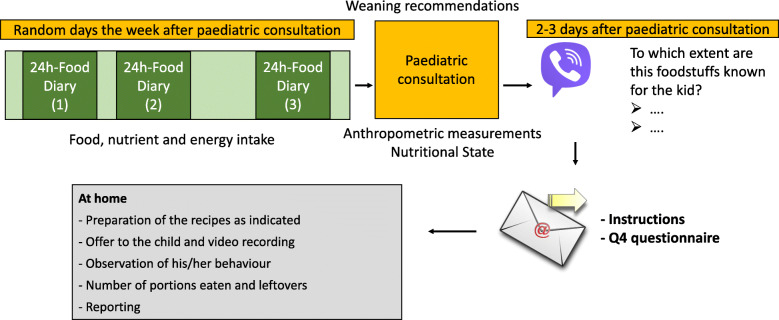
General procedure for the child observation events at 6, 12 and 18 months from birth

- Change of fruit and vegetable intake: measured by an ad hoc questionnaire reported by parents. At the beginning of the weaning process (depends upon each participant, 4th–6th month) and on the 12th and 18th month after delivery. Prior to the paediatric consultation date, each family will record during 24 h the food intake of the child indicating: the specific food consumed and its composition or commercial brand if necessary; the cooking method; and weight of the food consumed. Three days will be randomly selected within a week, ensuring that two of them are week days and one of them is a weekend day in order to avoid the “day” effect. Families will be provided with informative material on how to perform the measurements and record the information, together with blank forms they need to complete.
- Change of food acceptance to the exposed FV as the measurement of willingness to eat them: assessed by direct observations of child behaviour (see Fig. 2) and ad hoc questionnaires. Some days after the paediatric control visits on the 6th, 12th and 18th months parents will be contacted by telephone to gather information about foods which might be new for the child and have never been tried before. Any doubts arising on diet or possible recipes are clarified. Parents will be asked to record a video of these mealtimes and send them to the research team. Researchers will explain the video-recording protocol so that it is always recorded following a standard procedure. The Feeding Infants: Behaviour and Facial Expression Coding System (FIBFECS) [59] will be taken into consideration for the analysis of the video recordings. Swallowing times and acceptance or rejection signs will be taken as the main parameters.

#### Secondary outcomes

- Change of maternal diet during pregnancy and breastfeeding: measured by the Predimed questionnaire [60] and the Pre-coded Food Record Form [61]. The Predimed questionnaire has 14 items that evaluate adherence to the Mediterranean Diet, regarded as a good quality dietary pattern. The total score of the questionnaire ranges from a minimum of 0 to a maximum of 14 points. Each item has a different criterion to get 1 point, scoring positively if related to Mediterranean Diet. Thus, a total score of ≤7 indicates low adherence and a total score of > 7 indicates high adherence. We will use a validated Pre-coded Food Record Form to measure food intake in terms of portions consumed [61]. This tool contains the most commonly consumed foodstuffs for the Spanish population. The questionnaire is easy to complete and gives a precise indication of the foods consumed. Participants will fill in this questionnaire crossing out the corresponding boxes and indicating how many portions of each food or food group they consume in a certain day. It includes home examples for portion references in order to facilitate completion. Measurements will be taken at baseline, during the third trimester of pregnancy and after birth (except for the first month) every 2 weeks until the initiation of complementary feeding (4th–6th months after delivery, depending on each participant).
- Composition profile and rheological characteristics of maternal milk: measured by Infrared Spectroscopy MilkoScan (Foss Electric, Denmark), gas chromatographic- mass spectrometry (GC-MS) and rheometry techniques (rheometer AR1000, TA Instruments). Measurements will be taken during the 2nd month after delivery.
- Child’s nutritional state: assessed by the periodical paediatric control, reported then by the parents. Assessment will be carried out at the beginning of the weaning process, 6th, 12th and 18th months after delivery.
- Children Body Mass Index (BMI) from birth: it will be assessed by the periodical paediatric control, and reported by parents at 6th, 12th and 18th months after birth (following the paediatric calendar).
- Food allergen intake risk during complementary feeding will be assessed taking into account the procedures proposed recently in the scientific literature [62–64] and using self-developed tools for nutrient intake calculation [65].
- Mother’s psychological state regarding pregnancy: will be assessed by the Prenatal Evaluation Questionnaire [66]. This questionnaire is composed by 42 items divided in 7 dimensions: (1) pregnancy acceptance, (2) identification of the maternal role, (3) quality of the relationship with her mother, (4) quality of the relationship with her partner, (5) preparation for labour, (6) fear to pain and loss of control during labour, and (7) preoccupation for her and her baby’s wellbeing. Possible answers are: A = *a lot*, B = *with frequency*, C = *sometimes*, and D = *never*. Assessment will be carried out at baseline.
- Maternal anxiety: assessed by the STAI questionnaire (State-Trait Anxiety Inventory) [67]. We will only use the state dimensions in order to assess participant’s anxiety level at the moment the measurements are taken. It is composed by 20 items and answers are given on a 4-point Likert scale (0 = *absolutely not* and 4 = *very much*). Assessment will be carried out at baseline, on the 8th–9th month of pregnancy, on the 4th and 6th month after delivery.
- Level of depression: assessed by the Edinburgh Postnatal Depression Scale (EPDS) [68]. This questionnaire is composed of 10 items (answered punctuated 0–3 depending on symptoms severity) and will show us the level of the depressive symptoms of the participants. Assessment will be carried out at baseline, on the 8th–9th month of pregnancy and on the 4th and 6th month after delivery.
- Level of parental stress: assessed by Parental stress scale [69]. It has 17 items with a 5-point Likert scale from 1 = *strongly disagree* to 5 = *strongly agree* and it assesses levels of parental stress. Total score ranges from 0 to 63 and is obtained by the sum of the value for each item. Assessment will be carried out on the 8th–9th month of pregnancy and on the 4th, 6th, 12th and 18th month after delivery.
- Breastfeeding efficacy and certainty: assessed by the Breastfeeding Self-Efficacy Scale [50]. It has been used to predict breastfeeding duration and exclusivity. It has 14 items and answers are given on a 5-point Likert scale (1 = *not sure at all*, 5 = *completely sure*) and it is one-dimensional. Assessment will be carried out on week 2 after delivery and on the 4th month after delivery.
- Parental feeding styles: assessed by the Parental Feeding Styles questionnaire [70]. Its 27 items are divided in four subscales: emotional feeding, instrumental feeding, encouragement to eat new foods, and control over eating. Answers range from 1 = *never* to 5 = *always*. Assessment will be carried out on the 6th, 12th and 18th month after delivery.

### Statistical analysis

#### Quantitative data

The initial analysis of data will be performed on an *intention to treat* basis. A baseline descriptive analysis of the sociodemographic data and measured predictive variables will be carried out to ensure comparability between the intervention and control groups (mothers in the first phase and children in the second phase). Intra-group and inter-group differences in main continuous variables will be analysed by Student’s t test or non-parametric tests (Wilcoxon signed-rank test and Mann–Whitney U test) at different measurement times. For qualitative variables, comparisons will be made using Pearson’s chi square test.

Linear mixed-effects models with participant-level random intercepts will be used to study the effectiveness of the intervention and we will consider adjusting for any demographic or predictive variable results that show important differences at baseline. All statistical analyses will be conducted using the STATA 14.0 and IBM-SPSS 24.0 software. Confidence intervals of 95% and the significance level (*p* values) will be reported.

Subsequent to the initial analysis, we will also carry out a *per protocol* analysis in order to identify possible differences to the intention to treat analysis, which may occur as a result of a differential compliance of participants with the research protocol.

#### Qualitative data

The FIBFEC tool will be used for analysis of the video recordings on new food acceptance. Behaviours (reflecting avoidance or approach), facial expressions and rate of acceptance will be blind-coded according to the guidelines offered by the tool [71].

Spearman rho correlations will be performed to search for possible associations between coded variables. Descriptive statistics and two-way analyses of variance (ANOVA) will be used to test the differences in frequencies of behaviours and expressions between intervention and control groups regarding either the mothers' diet (SD vs. HFV) or the weaning method (SF vs. BLW).

### Ethical considerations

Volunteers must sign an informed consent before enrolment in the study, as well as other specific informed consent forms during the study, e.g. regarding children film footage and biological samples (i.e. breast milk). All participants will be free to leave the study at any stage of the project.

The present study has been approved by the Clinical Research Ethics Committee of the Basque Country (PI2019096) on the 25th of September 2019. It will be conducted following the rules of ethics stated by the Helsinki Declaration (52dn WMA General Assembly, Edinburg, Scotland, October 2000), the Standards of Good Clinical Practice and the current Spanish legislation regulating medical research involving human subjects (Royal Decree 223/2004 on clinical trials and Regulation 14/2007 on Biomedical Research). Data will be protected at all times to avoid not permitted use by external personnel. Confidentiality will be respected according to the Regulation (EU) 2016/679 of the European Parliament and of the Council of the 27th of April 2016 on the protection of natural persons (regarding processing of personal data and free movement of such data and the Law 41/2002, of the 14th of November, regulating patient autonomy and rights and obligations of information and clinical documentation. Thus, the data and information generated during the present study will be considered strictly confidential. Participants will be informed of the global results of the study.

## Discussion

The proposed project is a quasi-experimental study because authors consider that the characteristics of the sample and the independent variables do not allow a randomised approach. Authors believe that randomisation could cause problems such as, a low adherence to the assigned diet. Nevertheless, despite the lack of randomisation and with the objective of strengthening the study, steps proposed by the TREND statement for quasi-experimental designs will be followed. Primary care clinicians will help with the recruitment of pregnant women for the study, which will require an important effort to coordinate the recruitment process. Authors are aware that the management of a big (144 women) and heterogeneous sample size may be difficult. Nonetheless, simplifying measures will be set out and possible confounding factors will be controlled. In fact, a 20% dropout-rate has been estimated. However, possible losses when calculating the sample size have been taken into account.

Authors plan to observe children’s eating behaviour using video recordings during meals. In this regard, apart from the ethical implications of the information management (already considered in the project), it will be challenging to obtain well recorded, clear and usable video recordings. In order to facilitate the video recording process, families will receive precise instructions so as to ascertain the above mentioned requirements. We strongly believe that this methodology will provide stronger data as compared to the self-reported methodology, as previously observed by other authors [45].

This study looks for possible associations between the mothers´ whole diet profile, the physicochemical characteristics of maternal milk and food acceptance of infants. To the best of our knowledge, these aspects have never been studied before following the methodology proposed in the present study protocol. In addition to the mother’s diet, the relevance of a BLW approach will also be studied. Indeed, the relative weight of both factors, mother’s diet and weaning, will be characterised.

A relevant aspect of the present proposal is that, although it will be directed from the academic field, it will be executed in coordination with the Primary Care Services of the Basque Public Health Service (Osakidetza) and it will include the infrastructures and participation of the main Health Research Institutes of the Public Health System (Bioaraba, Biodonostia, Biocruces-Bizkaia). The question we would like to answer is: Is it possible to improve children FV intake tackling the mother’s diet during pregnancy and/or breastfeeding? And, is BLW a valid complementary feeding style that could be prescribed from primary care services? If the intervention is effective, it could constitute the basis for future public programs or nutrition guidelines aiming at improving children eating habits by increasing fruit and vegetable intake, which, could, in turn, result in potential health benefits.

## Data Availability

Data sharing is not applicable to this article as no datasets were generated or analysed during the current study protocol.

## Abbreviations

BLW: Baby-Led Weaning
BMI: Body Mass Index
EPDS: Edinburgh Postnatal Depression Scale
FV: Fruits and vegetables
SF: Spoon-feeding
STAI: State-Trait Anxiety Inventory
WHO: World Health Organization
